# Arteriovenous Graft Infection Due to Granulicatella adiacens

**DOI:** 10.7759/cureus.61622

**Published:** 2024-06-03

**Authors:** Sean D Delshad, Regan Ferraro

**Affiliations:** 1 Medicine, University of California, Los Angeles, Los Angeles, USA; 2 Radiology, Rolling Oaks Radiology, Thousand Oaks, USA

**Keywords:** infectious diseases, internal medicine, hospital medicine, avg infection, granulicatella adiacens

## Abstract

*Granulicatella adiacens* is a gram-positive coccus that is normally found in the human oral cavity and gastrointestinal and urogenital tracts but can rarely cause infection. When it does cause infection, *Granulicatella adiacens* has been most associated with bacteremia and endovascular infection, but to our knowledge, there are no previously documented cases of arteriovenous graft (AVG) infection. We present a case of *Granulicatella adiacens* bacteremia with associated AVG infection.

## Introduction

*Granulicatella adiacens* is a commensal bacterium of the human oral cavity and gastrointestinal and urogenital tracts [1]. *Granulicatella* species are gram-positive cocci that grow in pairs and chains and are catalase-negative [2]. *Granulicatella adiacens* rarely causes human infection, although data may be artificially depressed due to the historic difficulty of identifying *Granulicatella adiacens* on culture, as it requires pyridoxine or L-cysteine supplementation for optimal growth [3]. *Granulicatella adiacens* was historically classified as a nutritionally variant streptococcus until 16S rRNA gene sequencing demonstrated this group to comprise two distinct genera: *Granulicatella* and *Abiotrophia* [4].

Recently, *Granulicatella adiacens* has become a more frequently identified cause of bacteremia and endovascular infections in particular, especially in immunocompromised hosts [5]. There are reported cases of endocarditis, prosthetic valve infection, pacemaker wire infection, prosthetic joint infection, osteomyelitis, meningitis, septic arthritis, peritonitis, abscess, and empyema due to *Granulicatella adiacens* [6-9]. However, there are no previous cases of arteriovenous graft (AVG) infection due to *Granulicatella adiacens* documented in the literature to our knowledge. We report the first case of *Granulicatella adiacens* bacteremia with associated AVG infection.

## Case presentation

A 55-year-old male with a history of end-stage renal disease, hypertension, tophaceous gout, leukopenia, and total hip arthroplasty presented to the emergency room (ER) with fever and erythema and swelling at the left lower extremity (LLE) AVG site. The patient had a history of developing aneurysms and bleeding at various arteriovenous fistula and graft sites requiring multiple ligations and resections. Approximately six months prior to the current presentation, the patient had significant bleeding at the right thigh AVG for which he underwent a subtotal resection. At that time, a subclavian tunneled line catheter was placed for temporary hemodialysis access. Four months later, the patient then underwent construction of an AVG in the LLE, specifically between the left common femoral artery and the left common femoral vein. Two days prior to the current presentation, the patient developed fevers and chills as well as pain and swelling at the LLE AVG site. During scheduled hemodialysis, the patient had a fever with rigors and was transferred to the ER.

**Figure 1:**
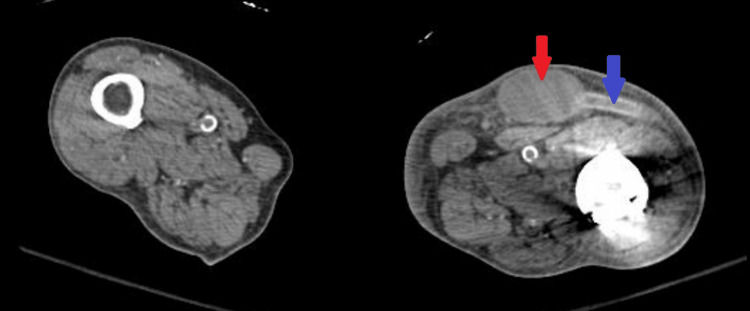
Axial computed tomography angiogram through the left proximal thigh demonstrating a moderate-sized intermediate-density fluid collection surrounding the left lower extremity arteriovenous graft, corresponding to the area of pain and erythema on physical examination Red arrow: fluid collection, blue arrow: arteriovenous graft

**Figure 2:**
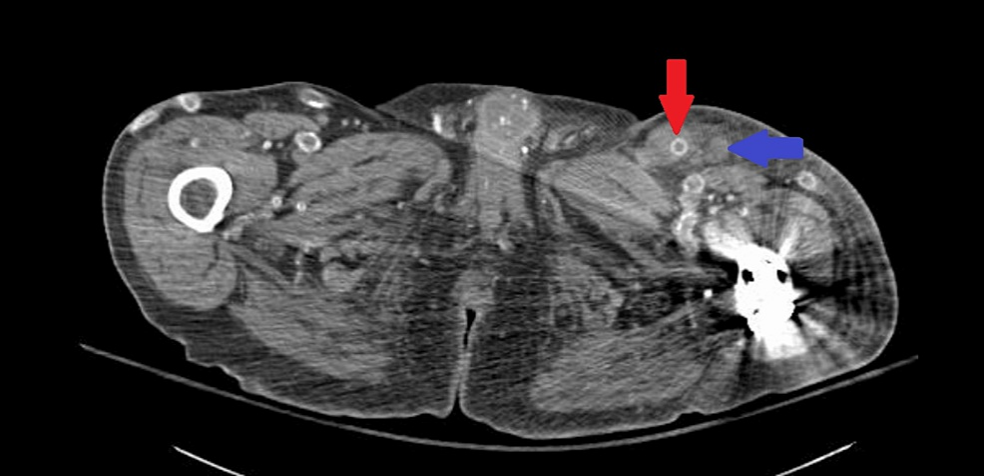
Axial computed tomography angiogram through the superior thigh demonstrating an intermediate-density fluid collection tracking along the venous limb of the arteriovenous graft with an adjacent reactive lymph node Red arrow: arteriovenous graft, blue arrow: lymph node

In the ER, the patient's vitals were a temperature of 40°C, blood pressure of 118/70 mmHg, heart rate of 119 beats per minute, respiratory rate of 18, and oxygen saturation of 97% on room air. On physical examination, there was warmth, erythema, and tenderness to palpation at the LLE AVG site. Examination of the oral cavity did not demonstrate obvious infection or poor oral dentition. Initial laboratory results were notable for a white blood cell count of 5,780/uL (normal: 4,160-9,950/uL) and a lactate of 8 mg/dL (normal: 5-18 mg/dL). Blood cultures were drawn, and the patient was started on empiric broad-spectrum antibiotics. A computed tomography angiogram of the abdomen and pelvis with lower extremity run-off demonstrated the LLE AVG with a moderate-sized intermediate-density fluid collection centered about the anastomoses, which tracked caudally surrounding the arterial limb, with trace fluid tracking along the venous limb cranially (Figure 1 and Figure 2).

Given the concern for an infected AVG, the patient urgently underwent excision and removal of the AVG. Intraoperatively, a gross visual examination confirmed the AVG infection. Postoperatively, the patient initially required vasopressors and underwent continuous renal replacement therapy via the subclavian tunneled dialysis catheter. He was continued on broad-spectrum antibiotics, intravenous vancomycin, and piperacillin-tazobactam, with gradual improvement.

Ultimately, the initial blood cultures grew *Granulicatella adiacens* susceptible to penicillin and vancomycin but resistant to cefotaxime. Blood cultures were positive within 24 hours on chocolate agar that was enriched with L-cysteine; there was no growth on blood agar. Cultures of the fluid collection surrounding the AVG and the AVG itself were negative. The patient underwent a transesophageal echocardiogram that was negative for endocarditis. Due to his history of vascular access difficulty, the existing tunneled line catheter was not replaced; instead, a strategy of salvage therapy was pursued. Given the presence of the pre-existing tunneled line catheter and the retained components at prior AVG sites, he was treated with six weeks of intravenous vancomycin therapy with the plan to continue with lifelong suppressive oral levofloxacin thereafter. The patient was ultimately discharged to a rehabilitation facility with the plan to follow up with his vascular surgeon to determine long-term dialysis access.

## Discussion

*Granulicatella adiacens* has been demonstrated to cause various infections, in particular bacteremia and endovascular infection. We have presented the first documented case of* Granulicatella adiacens* bacteremia with associated AVG infection. Our case further demonstrates *Granulicatella adiacens*' propensity to cause endovascular infections, including endovascular prosthetic infections. *Granulicatella adiacens*' ability to cause endovascular infection is likely due to its ability to bind to the host extracellular matrix and fibronectin [10]. Additionally, *Granulicatella adiacens* creates extracellular vesicles that allow biofilm formation [11].

Although cultures of the fluid collection and AVG remained negative in the presented case, AVG infection was confirmed based on the intraoperative findings. As the patient had already received intravenous antibiotics, these samples may have become sterilized. Additionally, given the fastidiousness of *Granulicatella adiacens*, growth on the cultures of the fluid collection and AVG may not have occurred due to the lack of the necessary nutrient enrichment. For example, the patient's blood cultures were positive on chocolate agar, but not on standard blood agar.

In the presented case, the patient's strain of *Granulicatella adiacens* was susceptible to penicillin and vancomycin, which is consistent with prior susceptibility data in the literature [3]. However, increasing penicillin resistance is of concern, as is resistance to later-generation cephalosporins. Furthermore, per the American Heart Association, susceptibility testing of *Granulicatella* species may not be accurate. In a study examining 109 isolates of *Granulicatella adiacens*, only 37% of the isolates were susceptible to penicillin, whereas 100% and 97% of isolates were susceptible to vancomycin and levofloxacin, respectively [12]. Given this susceptibility and resistance profile, empiric treatment with intravenous vancomycin is recommended when considering *Granulicatella adiacens* infection. In the presented case, despite the reported penicillin sensitivity of the patient's isolate, given the possibility of inaccuracies in susceptibility testing, the patient was treated with vancomycin as a review of the literature demonstrated significantly better susceptibility to vancomycin than to penicillin.

It remains unclear how the patient in the presented case developed this infection. Although a cursory examination of his oral cavity and dentition did not show obvious abnormalities, initial entry of the bacteria into the bloodstream was still most likely due to an odontogenic source. Proper dental care is an important aspect of treating and preventing further *Granulicatella* bloodstream infections.

The presented patient's infected AVG was resected, which is the ideal treatment to accomplish source control; however, given the patient had other retained prostheses and endovascular foreign bodies that could not be removed, a prolonged intravenous antibiotic course followed by chronic suppressive therapy was recommended. There are other documented cases of prosthetic retention and prolonged antibiotic therapy with success. For example, there is at least one documented case of a patient with a *Granulicatella adiacens* prosthetic joint infection who underwent debridement, irrigation, retention of the prosthesis, and prolonged antibiotic therapy with successful eradication of the infection [13]. Therefore, prolonged antibiotic therapy for infections caused by *Granulicatella adiacens* should be considered when prostheses or other foreign bodies cannot be removed or an attempt at salvage therapy is pursued.

## Conclusions

Although a normal commensal in the oral cavity and gastrointestinal and urogenital tracts, *Granulicatella adiacens* may cause various infections, most notably bacteremia and endovascular infections. As our case demonstrates, *Granulicatella adiacens* can also cause AVG infection. Given the propensity of* Granulicatella adiacens* to cause endovascular infection, patients with bacteremia due to *Granulicatella adiacens* and endovascular prostheses may require removal of the prostheses and/or long-term antibiotic therapy.
